# Hospital cybersecurity risks and gaps: Review (for the non-cyber professional)

**DOI:** 10.3389/fdgth.2022.862221

**Published:** 2022-08-11

**Authors:** Liat Wasserman, Yair Wasserman

**Affiliations:** ^1^Independent Researcher, Philadelphia, PA, United States; ^2^Independent Researcher, Boston, MA, United States

**Keywords:** cybersecurity, healthcare, hospital cyberattack, medical technology, data breach, patient safety, patient privacy

## Abstract

**Background:**

Healthcare is facing a growing threat of cyberattacks. Myriad data sources illustrate the same trends that healthcare is one of the industries with the highest risk of cyber infiltration and is seeing a surge in security incidents within just a few years. The circumstances thus begged the question: are US hospitals prepared for the risks that accompany clinical medicine in cyberspace?

**Objective:**

The study aimed to identify the major topics and concerns present in today's hospital cybersecurity field, intended for non-cyber professionals working in hospital settings.

**Methods:**

*Via* structured literature searches of the National Institutes of Health's *PubMed* and Tel Aviv University's *DaTa* databases, 35 journal articles were identified to form the core of the study. Databases were chosen for accessibility and academic rigor. Eighty-seven additional sources were examined to supplement the findings.

**Results:**

The review revealed a basic landscape of hospital cybersecurity, including primary reasons hospitals are frequent targets, top attack methods, and consequences hospitals face following attacks. Cyber technologies common in healthcare and their risks were examined, including medical devices, telemedicine software, and electronic data. By infiltrating any of these components of clinical care, attackers can access mounds of information and manipulate, steal, ransom, or otherwise compromise the records, or can use the access to catapult themselves to deeper parts of a hospital's network. Issues that can increase healthcare cyber risks, like interoperability and constant accessibility, were also identified. Finally, strategies that hospitals tend to employ to combat these risks, including technical, financial, and regulatory, were explored and found to be weak. There exist serious vulnerabilities within hospitals' technologies that many hospitals presently fail to address. The COVID-19 pandemic was used to further illustrate this issue.

**Conclusions:**

Comparison of the risks, strategies, and gaps revealed that many US hospitals are unprepared for cyberattacks. Efforts are largely misdirected, with external—often governmental—efforts negligible. Policy changes, e.g., training employees in cyber protocols, adding advanced technical protections, and collaborating with several experts, are necessary. Overall, hospitals must recognize that, in cyber incidents, the real victims are the patients. They are at risk physically and digitally when medical devices or treatments are compromised.

## 1. Introduction

### 1.1. The problem

With the emergence of cyberattacks in the 1970 s (1), cybersecurity has become a routine and major part of the technological world. Cybersecurity research has grown substantially in the past two decades (2), indicating the increasing concerns attackers present. The healthcare field, too, has been experiencing damaging security incidents. From storing patient information in the cloud to using artificial intelligence for radiology screening, medicine's growing reliance on technology is introducing innovative risks.

Already in 2011, a researcher managed to take control of and manipulate insulin pumps from afar, raising concerns that attackers can seriously injure patients. The US Food and Drug Administration (FDA) then recalled 465,000 St. Jude Medical pacemakers in 2015, following reports that the devices were susceptible to attacks (3).

Attacks are growing exponentially (4). By 2019, 24% of cyberattacks were in the healthcare industry (5). During 2014–16, 90% of hospitals and clinics experienced at least one data breach, and 45% experienced at least five data breaches (6). The number of healthcare breaches filed per year in the United States (US) has more than tripled in the past decade (7). Yet these are only the ones filed. There are, in actuality, many more data breaches than are reported because regulations require disclosure only of large-scale breaches—those affecting 500-plus records (8).

In 1 month at a United Kingdom (UK) hospital, 2.2% of emails and 2.9% of website actions were reported suspicious (9). These numbers may seem negligible, however, it takes only a single malicious email or activity to bring down a network. In 2018, a phishing incident at Baylor Medical in Texas resulted in the exposure of personal data belonging to 47,000 patients (10). That same year in Singapore, the medical information of the Prime Minister and 1.5 million other patients was stolen.

### 1.2. Research question

A question was thus advanced: are hospitals prepared for the risks that accompany clinical medicine in cyberspace? This study aimed to identify the current trends in healthcare cybersecurity according to a basic 4-point outline: (a) the healthcare cybersecurity landscape, (b) the major clinical uses of cyber technology and their security risks, (c) secondary risks associated with the technology, and (d) current strategies healthcare institutions have in place to combat the threats. Risks and strategies were compared to elucidate the security gaps.

## 2. Methodology

Articles were initially identified *via* search of the National Institutes of Health's *PubMed* database, which incorporates the *MEDLINE* database, due to access restriction for other databases and *PubMed*'s rigor and wide range of articles. The first article retrieval attempt utilized the keywords (cybersecurity AND healthcare) and resulted in 3,237 articles. Quotations were then added to narrow the search, (“cybersecurity” AND healthcare), and 154 results were produced. Preserving this keyword formulation, the search was filtered to include only articles published in English, peer-reviewed, and within the past 5 years (May 2016–2021) due to the constantly-changing nature of the cybersecurity field.

The search returned 132 results. Each article's title, abstract, and/or full article were manually reviewed *via PubMed* and Tel Aviv University Library's *DaTa* database, to ensure relevance of topic, non-duplication of articles, and availability of full text. Relevance referred to an article's addressing of the four outline points. The search concluded with identification of 32 suitable articles.

**Figure 1:**
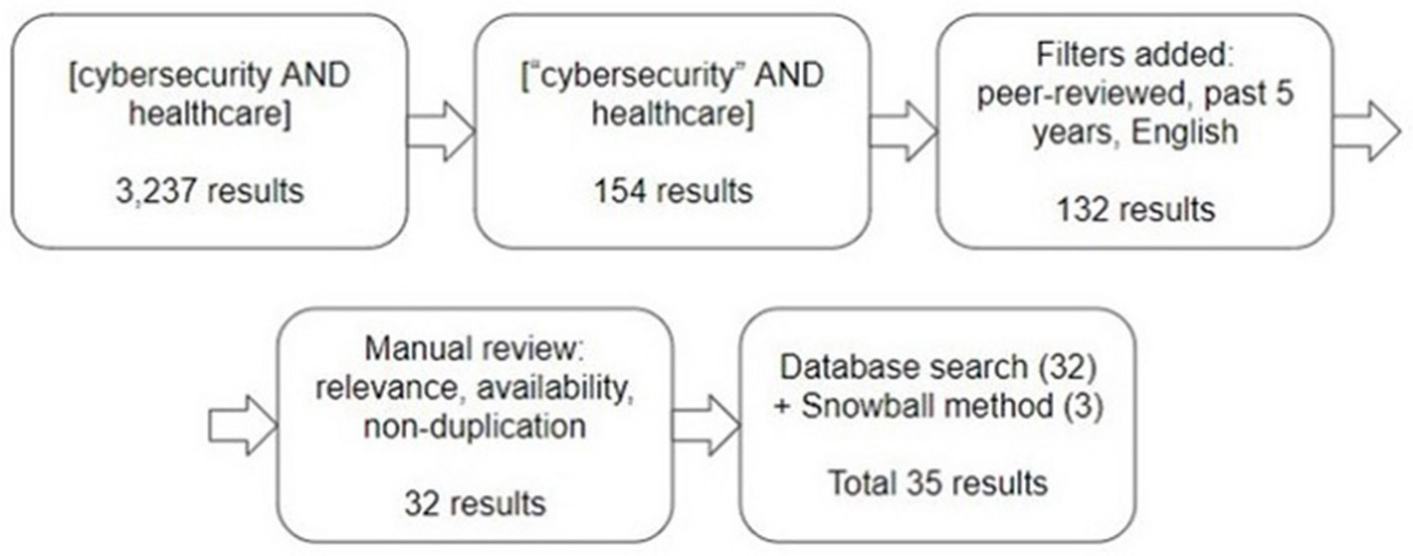
Literature review method.

A snowball method was simultaneously employed, *via PubMed* and *DaTa* or from the previously-selected articles' reference lists, to identify further manuscripts that fit the inclusion criteria. Three snowball articles that completely matched the criteria were selected for the final count. Thus, 35 articles, from mid-2016–2021, were incorporated to formulate the core discussion of this paper (see Figure 1 for illustration and Supplementary Table 1 for list of articles).

Articles were not excluded based on location, given the global nature of cybersecurity. However, as this research aimed to examine US hospitals' cyber-readiness, efforts were made to include only articles that were solely or including US-based authors (22 of the 35) for portions of the paper specifically describing American practices and recommendations. The 13 other articles were used to incorporate trends around the world. These international articles were from the UK (9, 11–15), Canada (16), Scandinavia (17), Europe (5, 18, 19), Middle East (20), and Asia (13, 15, 20, 21). Except for Middle East, all these countries scored “high” on the Readiness for Frontier Technologies Index of the United Nations Conference on Trade and Development's 2021 Technology and Innovation Report (22).

The 35 manuscripts were dissected and compared to determine the topics and concerns most commonly discussed in the literature. Ideas described by at least 2 authors were included, to ensure address of as wide a variety of experiences yet no solitary opinions. This paper represents the sum of these findings. Additional articles were then used to explain the core findings. Analysis was concluded inductively, with patterns of cybersecurity strengths and failures examined separately, then considered under the scope of real-world incidents. A second author reviewed and contributed to methodology, article selection, results, and analysis, to ensure quality and validity.

## 3. Literature review

### 3.1. The healthcare cybersecurity landscape

#### 3.1.1. Why attack hospitals

There are several types of actors involved in the cyberattack industry, including criminals, “hacktivists,” terrorists, spies, and ethical hackers, differing primarily by their goals, levels of credentials, and lawfulness. If these characteristics, especially the motives, of potential attackers are known, hospitals can better institute cybersecurity measures (23). Four primary motives were identified in the literature.

##### 3.1.1.1. Financial purposes

The most common motive of attackers is money (12, 24), accounting for 91% of data breaches (25). Each patient record is worth an average of $50 on the darknet (11), and a complete set of medical records can earn up to $1,000 (26). A social security number, in contrast, is valued at a mere $1 (26). Additionally, ransomed data is worth a lot, as it can also be sold to another criminal who will use it to extort the hospital again (27).

Stolen data can be used by hackers or their darknet customers to fraudulently apply for loans or other financial programs or receive identification (ID) documents (12). Patient ID data can, for example, be used to request free medical insurance coverage, like Medicare (24). Medical provider ID credentials, especially, can expand a hacker's access to the hospital network (27) or enable falsification of medication orders in order to sell the drugs on the darknet (12). As such, while other industry credentials are worth dimes (26), medical credentials are worth much more.

Criminal hacking is now more than a sport; ransomware, in particular, is now an industry (28). Attackers act as business owners, selling information or hacking tools to their darknet “consumers.” Some attackers even show market statistics like legitimate businesses do, including attack incidence rates, customer “success rates,” and standard prices requested for ransoms (28).

##### 3.1.1.2. Political purposes

Attackers may be acting on behalf of a political goal (12). During an international war, an attacking country may attempt to prevent the target nation from providing medical treatment to its citizens, harm the citizens by altering medical device operations, or uncover confidential information that can be used against the target country. Four percent of attacks are due to espionage (25). The offending group may also choose to attack for propaganda purposes. In 2017, the terror group ISIS hacked into the UK's National Health Service (NHS) website and posted images from the Syrian civil war, as part of its propaganda efforts (29). Cyberattacks committed by state actors and across borders are some of the most formidable. It is challenging to pinpoint and eliminate the attackers, and events often go unnoticed (11).

Alternatively, a hacker may have a domestically political motive. A 2014 incident at Boston Children's Hospital was initiated by attackers wanting to express resentment about the handling of a child custody case (3). A Romanian hospital's data was ransomed, as a statement against quarantine restrictions during the COVID-19 pandemic (13).

##### 3.1.1.3. Disrupt service

Criminals may act to disrupt healthcare services for the very purpose of disrupting services. Causing DoS, introducing ransomware, or infecting medical devices (30), for example, may be the end goal. Among other justifications, these attacks may be carried out for personal enjoyment, as are 5% of attacks, or may be in retaliation for a perceived slight on the part of the hospital or a physician, as are 1% of attacks (25). In fact, though this statistic has decreased recently, “vengeful employees” were identified in past years as some of the most likely to attempt cyberattacks (24). Overall, healthcare is impacted by cyberattacks more than other industries due to this ability to not only breach data but also disrupt operations (24).

##### 3.1.1.4. White hat actors

Hacking may also be caused by non-malicious actors. Though they represent only a fraction of hackers, some individuals, whether paid to do so or simply for fun, set out to discover vulnerabilities in hospital networks so that the issues may be fixed before malicious actors find them (12). Whereas malicious hackers are termed “black hats,” these good-hearted hackers are termed “white hats.”

#### 3.1.2. Common hospital attack methods

Cyber-attackers may target hospitals at any of three levels:

- Primary infiltration refers to an attack that directly impacts, maliciously or not, a hospitals' patients.
- Secondary infiltration occurs when the attack impacts the patients by implication only, not directly. Primary-level incidents may be strengthened by secondary level activities.
- Tertiary infiltration refers to a broader attack on a hospital's infrastructure, such as on supply networks, electrical grids, or economic management (31).

At any level, an attack generally follows a standard procedure. The malicious party first gains access to the network, possibly *via* unsuspecting system users. Once inside, the attacker assesses the system for what information or capabilities it has, specifically repositories of user account information, electronic medical records, medical device connections, and financial information, such as billing data. The assessment then turns to the databases chosen for infiltration, gauging their usual traffic and vulnerabilities. This is a critical step that will help the party enter, operate, and exit, while evading detection. The climax is then reached when the attacker targets the vulnerabilities and steals information from, shuts down, modifies, or impedes the network (32). The attack also opens new vulnerabilities, wherein the actor can later access further parts of the network (5).

The activities carried out in the last step can be classified as passive or active. Passive hacking means the attacker simply accesses and takes information, perhaps patients' information, healthcare providers' identities, or information about medical equipment. An active hacker deliberately pursues a system's functions, such as adjusting or stopping medical device operations or intercepting and modifying data collected by the devices (5). Both attack forms are expected to surge in coming years (5).

Houlding writes that the basic goal of cybersecurity is to ensure hospital data's Confidentiality, Integrity, and Availability (33). Each type of attack aims to compromise one of these elements. For instance, as will be described, ransomware acts to mess with availability of data (33). The review of 35 articles revealed ten types of cyberattacks generating the greatest concern in healthcare, with a variety of attack strategies used on hospitals (24). Attacks on healthcare networks are mostly “opportunistic,” going for those with easily-targeted vulnerabilities (33), and usually target a specific institution or group (24).

##### 3.1.2.1. Phishing

Phishing is a cyberattack manifested in the sending of a mass message, usually *via* email. Social engineering is exploited in an effort to influence at least one of the recipients to open the message, and either navigate to a website or download a file that has been rigged with malware (9, 23). Deception of the recipients often involves the message appearing to originate from reliable sources, such as peers or information technology (IT) employees (9).

In general, phishing is the most common delivery method (23) for offenders to infiltrate healthcare systems, with 89% of cybercrimes being initiated *via* phishing emails (27). The number of attackers who rely on social engineering has risen 25% since 2019, and phishing by itself accounted for 57% of healthcare cyber incidents in 2020 (27) – a sharp increase from 32% just 4 years prior (6).

Even in organizations considered to have strong cybersecurity, 30% of phishing attacks are successful (34), often due to the staff not recognizing the message as suspicious. Even higher rates are seen in less-prepared facilities. During 2011–2018, researchers sent out fake phishing emails to test how likely employees from six US hospitals were to fall for phishing emails. On average, employees clicked the infected links 14.2% of the time, essentially 1 of every 7 (35).

Within phishing, there are a few subtypes. Most infamous is spear phishing, in which messages are targeted toward specific recipients (23) to make it more appealing. Emails containing the malicious links or files are usually more personalized, increasing the likelihood that the recipient, often a senior manager, will fall for the attack. Spear phishing tends to achieve unauthorized penetration of a hospital's network just as much as general phishing (27). Clone phishing, which infects a credible email, and whale phishing, which focuses on high-level managers, are additional forms (9).

##### 3.1.2.2. Denial-of-service

Denial-of-Services (DoS) accounts for 48% of attacks (6). DoS involves actors “flooding a network with traffic” (23) to the point that the network is too overwhelmed to respond and thus cannot be accessed. Usually intended to ruin the hospital's reputation or physically harm patients (32), a DoS event can prevent medical teams from retrieving or sending patient data and can be expensive for the hospital to recover the network (23, 33).

A subtype, Distributed Denial-of-Services (DDoS) refers to DoS incidents that utilize several computers or other machines, usually internet bots, to perform the attack. More source computers enable a more formidable and incognito attack (36).

DDoS, and DoS in general, has been the cause of a number of widely-publicized cyber events. In 2014, a DDoS took Boston Children's Hospital off its internet network, including resources needed for patient care, for more than 2 weeks and resulted in $300,000 worth of damages (37). More recently, the US Department of Health and Human Services website experienced an attempted DDoS attack, just as people increasingly wanted to access the site during March 2020's COVID-19 outbreak (38).

##### 3.1.2.3. Privilege escalation

Privilege escalation involves converting a regular login account into an administrative one (32). Malicious software infects the computer, usually *via* phishing, and credentials are then taken from legitimate administrative accounts to add privileges to the target account. Administrative access can enable attackers to infect systems with more severe malware than regular accounts could achieve (32). A data management software, Philips IntelliSpace Perinatal, was found in 2019 to be vulnerable to privilege escalation attacks, which could be carried out by amateurs (39).

##### 3.1.2.4. Man-in-the-middle

Man-in-the-Middle (MITM) attacks occur when an unauthorized party exploits a vulnerability in the target party's network connection and surreptitiously inserts itself into the middle of communication transmission. The attacker can eavesdrop (40), steal, or modify information being exchanged before it reaches the receiving end of the communication (23). MITB, or Man-in-the-Browsers, is a relative of MITM wherein attackers infiltrate data exchanges from afar (23). Often in healthcare, MITM incidents lead to the leaking of sensitive patient information or manipulation of medical data, which can then be sold, repurposed to commit other cybercrimes, or even used to intimidate or extort affected patients (23).

##### 3.1.2.5. Malware

Accounting for 41% of cyberattacks in 2016 (6), malware refers to unauthorized software planted in a computer or machine that changes the activity or performance of that system contrary to the owner's determinations (32). Infection usually requires deceiving the computer's user into accepting malware onto the computer (32), usually *via* phishing (11) but sometimes *via* physical insertion (23).

Physical insertion of malware can be just as potent as phishing. Frequently mentioned in the literature are attacks in which infected USBs, external hard drives, or compact disks are “accidentally” left in employee parking lots. The expectation is that well-meaning staff members who find the devices will plug them into hospital computers to check the files and identify the devices' owners (23). Indeed, in an experiment by the US Department of Homeland Security, sixty percent of its employees who found devices in the parking lot inserted those devices into government computers. This number was higher, 90%, if the device carried a government or contractor logo (41).

Unlike DoS attacks, whose purpose is to shut down system usage, malware is usually intended to take control of a system for some time (32). The attacker can surveil, modify, damage, or erase sensitive data and activities on a hospital's network (23). Within the same category as malware, malicious activities can also be achieved with a virus, which is code that damages a computer's normal functioning and can spread between computers (42); worm, which is a malicious code that, unlike a virus, can operate and spread even without a host (23); Trojan horse, which is code that appears innocent and, though it cannot spread automatically, will infect and damage the computer once accepted onto the machine (43); bot, which is a software that can be programmed to automatically and quickly execute tasks, such as eavesdropping or spamming (44); spyware, which is software that observes and copies confidential data from an infected computer (45); and ransomware, as described below.

##### 3.1.2.6. Cryptographic

Cryptographic attacks, which enable hackers to surveil, steal, modify, delete, or otherwise damage patient records or other confidential information, can involve encrypting a hospital's data, decrypting it, or decrypting and then re-encrypting with another key (23). Oftentimes, hackers encrypt data to block access to its content until ransom is paid (46), commonly known as a ransomware attack. For example, ransomware targeted hospital computers and devices around the world in the 2017 WannaCry attack (described in “Consequences” section), an event regarded in healthcare as “one of the most impactful cyber-attacks in history” (3). In 2016, Hollywood Presbyterian Medical Center paid $17,000 to retrieve its data (3). That year, 44% of healthcare cyber incidents were due to ransomware (6). In 2020, hackers of Pennsylvania's clinical research company, ExecuPharm, re-encrypted files with new keys and demanded ransom. When the company refused to pay up, the attackers posted the sensitive data on public web pages (47, 48).

##### 3.1.2.7. Injections exploits

Also mentioned a few times in the literature are injection exploits, particularly SQL (Structured Query Language) Injections (23). A specific sequence of characters is inputted to specifically hit a system's vulnerability, resulting in destabilized or inaccessible system functions and potentially exposed data. This type of attack is usually done on internet servers or database systems (32). Few cases of hospitals experiencing injection exploit attacks have been reported, but the risk is evidently a topic of concern among cybersecurity researchers (23).

##### 3.1.2.8. Spoofing

Spoofing is a method in which hackers attempt to influence a medical device to receive an external signal, thereby allowing them to access or adjust the data, operations settings, and other system components (49). Spoofing is not difficult to do and does not require special tools (50). The most-used and most-effective method to hack portable medical devices is acoustic frequency matching, whereby the attacker tunes in to the device's frequency (49). One study, for example, modified data from the health tracking tool Fitbit using a speaker that cost a grand total of $5 (50). Another study utilized inexpensive infrared lasers to spoof an infusion pump's sensor (51).

##### 3.1.2.9. Destructive software

While typical malicious activities, like ransomware and malware, generally aim to observe, steal, modify, or encrypt information, a new form that surfaced in 2017, called NotPetya, aims only to destroy the files. NotPetya, and its relative Petya, is thus considered to be more problematic than the other damaging softwares (52). The software, determined by the US Central Intelligence Agency as having been released by the Russian military, destroyed systems belonging to some of Ukraine's financial institutions, power grids, airport, and governmental offices, essentially bringing much of the country's infrastructure, as well as some networks in the US, Denmark, and India, nearly to its knees (53).

##### 3.1.2.10. Drone-specific

A new attack method gaining momentum is the utilization of drones. Drones, also called unmanned aerial vehicles (UAVs), offer hackers the ability to be close enough to access almost any facility's network [current methods recommend that attacks be carried out within 10 meters (5)]. A small UAV was shown in two experiments to be able to be situated over hospitals, even ones in difficult-to-reach locations, and hack the networks without being noticed (49).

Drones attacks generally occur by first using a method of de-authentication wherein users are forced to disconnect from the network. Then, an “evil twin” attack is employed, wherein the drone presents itself as a genuine access point of the network, deceiving the users into logging into the drone's network. Following that, “wifi phishing” requires users to provide login information on an infected page before continuing to the network. At all three steps, healthcare providers' credentials are exposed to the attackers, so that they may now access the hospital's network and install malicious software, steal or encrypt data, or otherwise damage the system. This is known as a Drone-in-the-Middle (DITM), which is a type of MITM attack (49).

Drones can also assist in a “stepping stone” attack (49) during which drones, or other machines, are used to create communication chains between the attacker's and the victim's computers. The now-extended and complex system of message exchange makes it difficult for the attacker's computer to be identified or tracked (54).

#### 3.1.3. Consequences for hospitals

##### 3.1.3.1. Financial costs

Money is perhaps the most infamous consequence of cyberattacks on hospitals (12, 55). Twenty percent of attacks cause financial injury (27). Moreover, healthcare is the industry that spends the most money on dealing with data breaches, a whopping $7.13 million on average worldwide. In comparison, the average cost of data breaches in all industries worldwide is $3.86 million (56). Within the US, for the past 10 years that top-of-the-leaderboard status has been the same, and it is only getting more expensive. Costs have increased 10.5% since only 2 years ago (56).

Cyberattacks precipitate a slew of expenses: the cost of transitioning to emergency protocols, like recording patient data on paper instead of electronically, ransom fees, costs to repair or recover impacted systems, legal costs, public relations costs, costs of communicating the incident to patients, costs that result from loss of financial security, costs of abandoned medical appointments due to patient request or hospital need, costs of employing a workforce to deal with the breach, costs of changing or replacing the cybersecurity system including staff cyber education (57), costs of increased insurance premiums (52) and fines handed down as punishment from security oversight agencies. Additionally, each data breach disrupts and depletes customer trust, causing recovery from data breaches to be so expensive for the healthcare industry (56).

##### 3.1.3.2. Loss of data

Twenty-one percent of healthcare attacks cause data breaches (27). As opposed to non-medical information, such as financial material, when medical records are stolen or damaged, the data cannot simply be “reset” (11). Even if a hospital pays ransom for breached data, the attackers may still refuse to return the data until even more money is paid, return the data while also selling it to other hackers who ransom it again or to darknet customers who want the sensitive information, install malware or otherwise keep the systems infected in order to attack again in the future, return only some of the data, or return data that is different from the original set (58). All in all, per US privacy rules, hospital data that has been ransomed or otherwise attacked is considered unreliable, or at least permanently compromised (59).

##### 3.1.3.3. Reputation and trust

While financial loss incurred by a hospital due to cyberattacks is one of the most publicized consequences, one of the most damaging is the negative effects on the hospital's reputation (55). Data breach can inculcate a sense of distrust between patients and healthcare providers (30). Decreased trust will, in turn, make patients less likely to share personal information with providers, including information that may be clinically significant (11, 12). The fact that an estimated 67% of hospitals do not have programs in place to assist patients whose data has been exposed (6) can further damage trust relationships following an attack.

##### 3.1.3.4. Physical harm

A troubling potential consequence is physical harm to the patients (12, 30, 55). Fifty-five percent of attacks in recent years interfered with hospitals' networks and services, and 18% interfered with or damaged systems necessary for medical care (27). Incidents have caused critical patient injury (27). At the ancillary level, digital hospital equipment, like computer-run elevators needed to transport patients or lab samples and computer-run HVAC systems needed to maintain sterility in operating rooms, can be shut down or made to malfunction (27). Additionally, resources will need to be reassigned to deal with the attack, so less resources will be available for medical care during the event and recovery (60).

More directly affecting patients, both in and out of the hospital, cyberattacks can target medical devices. In March 2019–2020 alone, the FDA sent out five distinct alerts regarding cybersecurity vulnerabilities in routine medical devices (61). Medtronic's insulin pumps, for instance, were recalled because it was found that third-parties could wirelessly access the pump's remote control and adjust the therapy. If attackers shut down the device or reduced the dose, patients could enter a state of hyperglycemia, diabetic ketoacidosis, or death. On the other end of the spectrum, if an attack triggered excessive doses, hypoglycemia or death could be induced (62).

Implantable devices are especially at risk because surgery or invasive procedures are required to replace the devices should cyberattacks damage them (16). St. Jude Medical's Merlin@Home Transmitter for controlling implanted cardiac devices was an example. A hacker could prevent data from being forwarded to healthcare providers, stop the connected devices from operating by inducing malfunctions or running down the battery, or induce a pacemaker to speed up its rate (5), all of which could cause serious medical emergencies.

The severely damaging WannaCry attack on the UK's NHS (3) showed the practical consequences on clinical care. Patients needed to be transferred to other facilities, and ambulances needed to be redirected to other hospitals. This harmed many patients, including those with time-sensitive emergencies who were now rerouted to more distant facilities and those needing procedures, including critical ones like open-heart surgeries. Also, refrigerators for critical supplies were locked electronically as part of the attack (14).

Cyberattacks can diametrically affect hospital clinical outcomes (60) in the constant struggle between security and usability/availability. In the US, from 2011 to 2017, 30-day fatality rates for acute myocardial infarction (AMI) dropped on average 0.4% each year. But in hospitals where data breaches occurred, the 30-day fatality rates for AMI did not just not decrease, but they actually increased 0.34–0.45% per year for 2–3 years. To explain the trend, the researchers postulate that these differences may have been due to data breaches driving the hospitals to institute new cybersecurity protocols that were too novel for medical providers, thereby exasperating them and causing mistakes (63). No matter the root cause of the statistics, the study is unambiguous in its message of patient safety.

##### 3.1.3.5. Extended effects

Attacks on one institution can have global effects on others. In 2012, a single phishing email managed to temporarily take down the Saudi Aramco petroleum and gas corporation. In order to recover, the company tossed the infected hard drives and bought 50,000 new ones. This caused the price of hard drives to increase worldwide for 5 months, in addition to causing delays for hard drive suppliers (64). A cyber event in one hospital may similarly affect other hospitals' supplies, operations, or cyber vulnerability.

### 3.2. Current cyber clinical technologies and their security risks

#### 3.2.1. Medical devices

##### 3.2.1.1. The technology

Medical devices can be categorized according to their purposes: (1) diagnostic, (2) monitoring, and (3) therapeutic (65). Diagnostic devices are used in order to identify a patient's medical state, such as determining the cause of a patient's symptoms. This category includes ultrasounds, EKGs, pathogen identification test systems, and more.

The most common category, monitoring devices (66), provide continuous observation of a patient's health, alerting when physiological indicators deviate from baseline values. Examples include ventilators, cardiac monitors, pulse oximeters (49), remote activity monitoring for persons with dementia (67), and independently-selected health trackers such as FitBit (49).

Therapeutic devices provide treatment to a patient when the need arises. Many such devices are externally connected to the person, and some are implanted. Most commonly implanted are cardiology instruments, such as defibrillators, pacemakers, and cardiac resynchronization therapy systems (5). Other major therapeutic devices include insulin pumps for diabetes, dialysis machines for kidney failure (5), and deep brain stimulators for seizures and Parkinson's disease (68).

In recent years, medical devices have become electronically connected to networks to allow for continuous monitoring (5), and clinicians are transitioning more and more to the use of remote web-connected devices. The tools allow patients to be treated from home instead of needing to visit the hospital, saving time and resources for patients and hospitals alike. If emergencies arise, providers can quickly assess (5), direct, and potentially treat (69) the patients remotely. Remote devices have also been shown to improve clinical care. Patients fitted with cardiac implanted devices that physicians monitored from afar had 45% decreased mortality compared to patients who relied on in-person appointments for monitoring (70).

As the rate of chronic conditions increases (71) and individuals place more focus on health, the number of remote medical devices, also called Wearable Internet-of-Things (WIoT), has been surging (69). In fact, the WIoT market dollar value is growing by the billions (66). Globally, 7.1 million patients utilized remote medical monitoring devices in 2016, and 50.2 million are expected in 2021 (72). In the US, 2018's 1.8 million healthcare WIoT devices distributed is expected to jump to 6.9 million in 2023 (73). Currently, 30% of adults in the US utilize wearable medical devices (74). It is thus crucial to understand the technologies' risks.

##### 3.2.1.2. The risks

Some have posited that medical devices may be at less risk than other hospital equipment. One argument is that hacking medical tools requires basic skills plus familiarity with such specialized devices and, many times, understanding of the clinical effects of device modifications (5). In other words, as infiltrations are usually for stealing data or harming patients, and not to simply disable devices, hackers would need to be aware of where and how to change the device to induce the intended effects. Familiarity and clinical proficiency are rare in hackers and would therefore reduce the likelihood of attacks (5).

This argument is counteracted by the multitude of experiments that have succeeded in hacking devices and creating issues of data privacy and physical safety (51) with minimal difficulty. Already in 2008, researchers managed to infiltrate and manipulate implantable defibrillators with simple radio waves (3). Other researchers succeeded in spoofing sensors of typical infusion pumps used for critical patients (51), some even bypassing the need for internet access that spoofing generally requires (21).

The second argument is that some medical devices and their settings cannot be modified remotely (5). However, this does not take into account that patient data can still potentially be accessed from afar, with the device being left undamaged and without knowledge of the access. For example, a smartphone application connected to a device can be infiltrated, leaving the device unimpacted and unaware of a data theft. Additionally, systems used to reprogram medical devices can be bought in public markets, like Ebay, or simply taken from medical clinics, where the systems are sometimes not locked away. Many do not require passwords nor have encryption, so they can easily be hooked up to manipulate devices (75).

In effect, medical devices are actually more at risk for cyberattacks than other technologies. Medical devices provide ample opportunity for hackers to access data, adjust patient care (5), or further infiltrate a hospital's network. Seventeen percent of attacks succeed in infiltrating the networks *via* medical devices (76).

One reason for the increased risk is that the security of many devices is not up-to-date, as they are designed to last more than 5 years. Within 5 years, the cybercrime landscape can shift dramatically and be many steps ahead of the devices' security that had been set years before (77). For instance, a device (Medtronic implanted defibrillator) used to require a maximum two-inch distance to be hacked (75), but in 2016 researchers spoofed a device (an infusion pump, this time) using cheap tools at a 12-meter distance. If the tools were higher quality, the attacks could have succeeded even farther away (51), something for which the Medtronic defibrillator would not have been prepared.

A second reason can be attributed to the changing legislative landscape. Beginning in 2018, the US Centers for Medicare & Medicaid Services offered reimbursements to healthcare providers who fitted their patients with devices that allow real-time data transmission (78), incentivizing the increase in permanently web-connected monitoring devices. Yet, devices that only transmit data when the care provider and patient initiate transmission and reception simultaneously, as was the process in the past (75), or devices that automatically connect to the internet only at specified intervals still carry risk during those connection times (5). Devices that are always connected are thus at even higher risk.

Relatedly, although not themselves medical devices, smartphones that connect to devices or otherwise interact with patient data are a third cause for increased risk. Smartphones are gaining traction as a method for controlling medical devices, such as insulin pumps (75). Also, some smartphones include biosensing capabilities. During COVID-19, for example, phones could be set up with pulse oximetry sensors to assess severity of COVID-19 illness. This feature was determined to be adequately reliable to qualify for FDA certification (79) and therefore may have been used by physicians to evaluate patients remotely. Smartphones lack sufficient protection and carry high risks of infiltration, malware, MITM, and others. Other health-related apps that log patient data, such as medication management apps, are also now ubiquitous (18). However, they are often insecure, lacking even basic mechanisms like passwords and encryption, thus potentially compromising medical records (80, 81).

In any case, recent advancements in hacking methods are beginning to enable attacks whether or not a device is connected to the internet, a fourth reason for medical devices' increased risk. Previous generations of devices transmitted data *via* telephone or cell tower, so hacking was more difficult (75). Nowadays, attacks can be carried out *via* radio waves, Bluetooth, and other non-internet based tactics at any time (75). Some of Medtronic's pacemakers and defibrillators, for example, can be manipulated with simple magnetic fields (21, 82).

A last, yet significant, reason medical devices carry higher cyber risk relates to the device companies. Commercial products intended for professional medical utilization emphasize “functionality” rather than cybersecurity (58). As of 2016, just 2.13% of medical devices were accompanied by descriptions of their cybersecurity features. This number has increased in recent years, but not nearly enough (76). Non-specialty devices were 22.2% likely to have mention of cyber protocols, but ones for orthopedic, surgical, urological, and gastroenterological specialties had no mention of cybersecurity in their summaries. Lack of security-focused manufacturing and merchandising can prevent awareness and adherence to safe cyber activities by both patients and providers (76). Additionally, many devices are created so that they can only be patched or monitored for vulnerabilities by their manufacturers. As such, despite 90% of attacks occurring because of system errors on the part of the developers (23), hospitals cannot apply their own security risk-reduction measures (13).

#### 3.2.2. Telemedicine

##### 3.2.2.1. The technology

Even before COVID-19, telehealth was gaining momentum as an integral part of healthcare. Telehealth refers broadly to the interaction of medicine and digital technology (83). Specifically, telehealth includes doctor's appointments *via* visual-audio platforms, treatment in which a provider is instructing from afar, medical training for providers and patients *via* internet platforms, and web communications or data exchange regarding health matters (21).

In the program through which telehealth is executed, there are two parts: the platform and the wider system. The platform is what is available for use by the patient and healthcare provider, whereas the wider system is what is available for use solely by the healthcare provider and medical peers (21). The platform is therefore often operated on insecure computers, whereas the rest of the system is more likely opened on hospital security-approved computers. At the same time, many of the risks are on the hospital's end, less so on the patient's, as the hospital's side encompasses more sensitive data access sharing, like sending drug prescriptions. The medical provider's end therefore needs better cyber protections.

##### 3.2.2.2. The risks

There are several potential sources of cyber risks in telehealth. First, the patients, especially in older populations, do not usually know how to protect from cybersecurity threats on their end. When using the telehealth system, they may have easily-guessed passwords, accidentally expose their device or the telehealth software, fall victim to phishing attacks, or end up misplacing the device connected to the telehealth system (and by extension the sensitive information) (21).

Second, the computers used most likely have third-party applications through which the telehealth platform is open to infiltration. Hospitals may maintain devices that are set up only for specialized activities that require use authorization. Should a secure telehealth program be used on this computer, the system will generally be safe from infiltration on the hospital's end (21). However, other hospital computers, which host more than just this software program can be more easily infiltrated (21) because there is a higher chance for vulnerabilities somewhere on the device.

Third, the gateway devices used between the system and patients are at risk. Gateways communicate wireless internet to reach computers and are like front-doors to one's private network (84). Attackers can perform MITM attacks, steal the gateways, or create “rogue gateways” (21), essentially masquerading as the real gateways to intercept data without authorization. The internet itself that is connected on either end of the system is similarly at risk. Internet exchanges, usually done wirelessly, are not private and are thus penetrable. End-to-end encryption can mitigate penetration, but any vulnerabilities can open the telehealth system to data interception, eavesdropping, and manipulation, and eventually possibly to attacks of privilege escalation (21). Then, the network connected to the vulnerable internet is also open to MITM and data interception (21).

Fourth, the telehealth software, which contains a plethora of patient information and includes a method of interfacing with patients, can be infiltrated. Attacks may be with MITM or malware, but could also lead to manipulations of the telehealth software itself or be a stepping stone to the hospital's wider network. If the company providing the software service is not secure, threats may also include manipulation of medication prescriptions, sensitive information exposure, eavesdropping, or malfunctions in the computer connected to the software (21).

#### 3.2.3. Electronic data

##### 3.2.3.1. The technology

Health information is one of the most confidential datasets that exist (20). The Health Insurance Portability and Accountability Act (HIPAA) of 1996 recognized this and mandated protections for sensitive health information. Therefore, demographic data, medical and mental history, test results, insurance details, and information providers need in order to care for patients are protected under the law (31).

One requirement of HIPAA is that data breaches of more than 500 patient records (85) must be reported and patients informed (60). So large a breach was unlikely, until electronic medical databases came into being. At that point, further security standards were necessary to manage the novel risks cyber technologies were creating. The 2003 HIPAA Security Rule established security requirements for electronic patient data. Six years later, in 2009, the Health Information Technology for Economic and Clinical Health Act (HITECH), encouraged the use of electronic medical records and increased HIPAA security requirements (3). No matter how strict they are, though, HIPAA protections are constantly circumvented by attackers (1).

##### 3.2.3.2. The risks

Risks are specifically seen with regards to data located on patient portals (20), which are often accessed from patients' computers with a lack of cybersecurity practices precautions, and electronic medical records (EMRs). Taking the place of paper records, EMRs can improve treatment, make access to patient records easier, help patients communicate with providers, enable patients to take control of their health and data, and overall save money (58). According to the CDC, as of 2017, 80% of physicians (86) and 96% of hospitals (87) used certified EMRs. However, any improvement in web accessibility of records for authorized parties also means improvement in accessibility for malicious parties. Compared to paper versions, electronic data is easier to steal undetected (12). Digitalization offers monitoring of access to it, but hackers know how to get around this monitoring (12).

As a note, as opposed to EMRs, electronic personal medical records (PMRs), which may be provided by medical institutions and are maintained by the patients themselves (88), are not are bound by cybersecurity regulations, like HIPAA and HITECH (24). Providers and patients must be aware that PMRs carry higher security risks, as data entered into the PMRs may offer hackers enough information to access other sensitive records.

The same threat is present for data storage. In the past, hospitals mostly maintained sensitive databases and devices within an intranet (on-premise), and not connected to the external internet. This made it nearly impossible for outside infiltrators to gain access (33). Now hospitals are moving toward cloud-based computing, which essentially means operating computers and networks *via* web-connection (89). Using the cloud for data storage (20) is less expensive, provides increased data accessibility, and offers increased sharing capabilities (33). At the same time, connection to the internet, even if it is a private cloud (89), opens the hospital to cyber threats. As long as there is a web connection, hackers can infiltrate (18).

Relatedly, “Picture Archiving and Communication Systems” are software for medical imaging-based clinical diagnoses (18). In this category are X-ray, MRI, and other radiology data storage systems (90) and, indirectly, advanced technologies like artificial intelligence programs for radiology readings. The systems carry the same risks as other data storage and transmission tools, and there is an added concern that attacks can manipulate the images, causing diagnoses to be applied incorrectly.

### 3.3. Associated hospital cyber vulnerabilities

#### 3.3.1. Interoperability

Each US hospital bed is monitored by 10–15 cyber-connected devices on average (91), with more expected in coming years (1). Fernández Maimó et al. call this an “integrated clinical environment” (46). As a result of the 21st Century Cures Act passed in 2016, the integrated health system further refers to the sharing of information between hospitals (92). This capacity to exchange information between multiple devices and organizations creates significant concerns, termed “interoperability” in the literature.

From treatment apparatuses to staff personal phones (35), interoperability is advantageous for patients and providers alike. Patient data is shared more easily, which means more coordination among the members of the health team. The patient can be monitored more comprehensively, so less face-to-face appointments or invasive treatments are needed, and multiple electronic inputs can provide a more accurate and methodical set of patient data (12). For example, if each hospital department with which patients interact, from nursing stations to laboratories, were able to connect and add information to patient records, then clinical care would be better managed (35).

Nevertheless, interoperability also leads to more cyberattacks (60, 92). The multiplicity of interconnected devices increases the probability that at least one device will contain a vulnerability, creating more potential infiltration points (92). Infiltrating one device can enable access to the entire system, and, by extension, the entire database of patient information, to which that device is connected (12, 92).

#### 3.3.2. Out-of-date operating systems

Hospitals' tendency to rely on unprotected computer operating systems is a major vulnerability (12, 60), according to researchers. As of 2019–20, 71–80% of Windows computers in hospitals were using old, unsupported versions, like Windows 7, 2008, or XP (27, 93). Unsupported systems tend to carry more vulnerabilities, as the manufacturer does not add protections to these versions while cyber-attackers develop more advanced and potent methods. Similarly, some hospitals stick with old security measures, including 11% still working without firewalls and 9% without even basic antivirus or antimalware (27). Most do not have adequate recovery protocols, in case of attack (27).

#### 3.3.3. Lack of resources

Network vulnerabilities may also be attributed to weak hospital cybersecurity departments. Seventy-three percent of healthcare organizations are incapable of managing cyber incidents (4). Most IT departments do not run complete risk evaluations of the networks (27), with only 16% scheduling evaluations of system vulnerabilities more than annually (6). Twenty-nine percent reported not having cyberattack response plans whatsoever (6), and of those who do, 80% have not actually tested their cyber incident protocols (4). The numbers gain more significance when comparing to other industries. The average time it takes for all industries to identify and then manage data breaches is 207 and 73 days, respectively. The healthcare industry takes the longest to identify and manage breaches, 236 and 93 days, respectively (56).

An underlying cause for weak IT departments is inadequate resources for the teams. To start, the departments often are inadequately staffed (23, 60, 94), with 1.8 million too few IT employees predicted by 2022 (95). Thirty percent of healthcare staff believe IT departments are responsible for managing cyberattacks (6), but a lack of cyber professionals, possibly due to hospitals offering low salaries or not being driven to hire them (23), will mean there is no IT team to fill that role. Additionally, cybersecurity involves multiple fields, from IT to privacy departments. Ambiguity with regards to who is accountable for the security can mean no one takes the responsibility (11).

Another resource missing is money, and by extension, ample security equipment. Fifty-six percent of hospitals reported insufficient cybersecurity resources and budgets, and 40% reported needing external cyber experts to assist in responding to breaches (6). Seventy-five percent of hospitals believed not enough funding was allotted for cybersecurity, with only 40% believing adequate funding would be provided in 2021–23 (96). Despite constantly increasing risks, IT budgets were reportedly reduced or maintained at constant level in 62% of hospitals (6).

Of the IT funding, cybersecurity usually makes up <6%, preventing the purchase of up-to-date security resources (27), which are often fairly expensive (11). Governmental agencies have set standards regarding the level of security measures healthcare networks must maintain, as discussed in “Regulatory Measures”, but most hospitals do not have adequate resources and time to comply with requirements (28).

#### 3.3.4. Focus on medical care

One reason the budgets do not include much money for cybersecurity purposes (12) is that healthcare organizations tend to prefer to direct resources to clinical care, inevitably leaving cybersecurity by the wayside (97). This neglect was highlighted by the Department of Health and Human Services in 2017, as healthcare is one of the few industries that have not incorporated security into their operations (60).

#### 3.3.5. Rapid technological innovation

Healthcare is constantly evolving in its technology. Radiologists can now use artificial intelligence to read slides (98) and nurses can be assisted by radio waves to ensure they are providing treatment to the correct patient (20), for example.

COVID-19 drove digital advancement of healthcare, such as telemedicine, at an even faster pace (13). With this advancement, cybersecurity and privacy considerations were often neglected (99), and opportunities for attacks proportionally increased (15). During pandemics, HIPAA is not enforced as strongly so that hospitals can take advantage of less-secure but necessary health resources (55). The COVID-19 experience showed how advanced technologies need better cyber protections.

Each new technological advancement can save lives, but each one can also be a new method for cyberattack (31). This is the often-cautioned “cyberthreat paradox” (100). Nevertheless, in a German study, 93% of hospital workers believed that the benefits of healthcare digitalization are worth the risks (30).

#### 3.3.6. Constant accessibility

Attackers are aware that hospitals possess large amounts of sensitive data (1, 30) and that the data must be readily accessible. This causes some vulnerabilities.

First, medical data and health networks must be connected and accessible to healthcare providers at all times, every day (58). Many people often need urgent access to the data. To illustrate, during medical emergencies, secure and prompt communication is needed between the patient, family members, pre-hospital first responders, hospital healthcare providers, and hospital administrators (20). The network is constantly open to threats, and a slip in cyber protection vigilance at any time can invite a devastating attack.

Second, hospitals often give in to ransomware attacks and pay the fee to retrieve their data because of the urgent need to access medical records (101). The Federal Bureau of Investigation does not condone but does seem to acknowledge that many have little choice but to pay. The encrypted keys to access the ransomed data are nearly impossible to guess or bypass (28), so the only options are to lose the records or pay. An organization that pays ransom, though, is marked by the attackers as one that will give in. Attackers will be more likely to strike again (28), and other hospitals will be put at risk, as well, as hackers learn they can easily extort healthcare organizations.

Third, the drive for constantly accessible data means that outside devices are now being used in addition to secure hospital devices. Personal computers, especially for employees working from home, and individual devices, such as smartphones, have access to patient data (12). Additionally, outside devices belonging to third party contractors may be introduced into the hospital system. HIPAA applies only to contractors if they will be dealing with sensitive medical information (57). Contractors who are not involved in sensitive data matters may still connect to the network, bringing their unprotected device vulnerabilities with them (57).

#### 3.3.7. Internal threat

Cyberattacks in healthcare are largely enabled by internal employees (25, 102). Unaware employees made up 40% of actors that induced cyber events (27). 27–35% of attacks in 2020 were due to human error (56), usually as employees failed to adhere to hospitals' cybersecurity protocols or unintentionally clicked on phishing links (103). The former can be the result of staff not being used to or not understanding tight cyber protections, or the result of healthcare providers focusing more on patient care, at the expense of cybersecurity (103).

Unintentional clicking on phishing links is likely the result of inadequate training. Among the interconnected network of devices, applications, databases, users, IT teams, and more, if a component lacks sufficient protective measures, attackers can infiltrate the system by exploiting that weak point (23). Medical providers connecting to the network can thus invite cyber threats if they are not proficient in privacy regulations and security protocols (23). Fifty-two percent of IT teams who experienced cyberattacks felt that better cyber education for staff was necessary to prevent future events (6).

The high statistics are unlike in any other industry (97). Hospitals are particularly vulnerable to internal threats because frequent employee turnover is prevalent. There are continuously new staff that need to be trained in cybersecurity principles (35).

Of the 23% of phishing incidents that were due to insiders in 2020, 13% were attributed to unintentional activities. The other 10% were attributed to malicious actors (27). The “malicious insiders,” who constitute 17% of all actors (27), are employees who purposely assist the attackers (23), whether for money or as revenge. Data from 2019 showed that 39% of attacks were internal (25), whereas 2020 data estimated it to be around 30% (34). As tests to assess hospital employees' security knowledge have not shown improvement in results, the decrease in internal threats may be due to less malicious employee attacks (25).

#### 3.3.8. Lack of regulation

When the FDA began taking notice of medical device cyberattack risks in 2015 (5), the agency created the National Evaluation System for Health Technology. The intention was to enable faster evaluations of medical devices (75). FDA evaluations, like those for drug safety, usually take long. The ever-changing landscape of the healthcare cyber world necessitates more immediate actions.

Despite the evaluations, the availability of a plethora of cyber technologies, especially EMRs, makes it difficult to actually regulate them. Medical equipment producers, for example, are not included under HIPAA laws (92). As such, the FDA assigned responsibility for cybersecurity of medical devices to the manufacturers themselves and relies on them and the hospitals to ensure safe cyber practices (12). The statistics in “Associated Vulnerabilities” section regarding institutions' cyber protection practices give the sense that such trust may not be warranted.

### 3.4. Current hospital cybersecurity strategies and their gaps

#### 3.4.1. Technical measures

Current cyber protection actions undertaken by hospitals most often relate to security on the user's end, be it the medical provider's and/or the patient's end. Some actions involve allowing navigation only to specific websites, requiring difficult-to-guess and regularly changed passwords, and allowing connections to the hospital network only by facility-approved devices (60). These specifications are intended to make computers, accounts, and networks less vulnerable, but they are often not enough to prevent breaches (104). For example, the thirty most attacked vulnerabilities in 2015 were actually password-independent (105).

On the IT department's end, active steps taken include segmentation and patching (60). Segmentation refers to separating the hospital network, including the devices connected to them, into small sections. Even if a malicious actor succeeds in infiltrating one of these sections, the others remain secure (106). Similar to using cloth patches to cover holes in clothing, patching is a method of covering software vulnerability “holes.” When a vulnerability is found in a system's code, the manufacturer will usually release a patch, sometimes in the form of a program upgrade (107). IT departments themselves are also often on the lookout for vulnerabilities in their systems.

Relying on updates to add protections is not completely effective, particularly for medical devices. Updates can cause the device to stop working, reset the device settings, or may not even succeed in updating to add the cybersecurity features (75). In fact, due to the risks associated, some patients refuse to update their medical devices. And regardless of IT professional recommendations to update, half of providers fail to do so to their clinical equipment. Thirty-nine percent stated that they did not update because they were concerned the device would stop working, and 23% stated they did not believe updates were important (75).

Hospital computers can also be set up with a variety of protective software, most commonly antivirus and antimalware programs. Nevertheless, such software generally works according to attack trends. They are unable to catch ransomware attacks that appear or operate unconventionally or that infiltrate undetected, such as by appearing like the regular traffic running on the device (46). Additionally, IT departments may choose to use detection software that are less sensitive, and thus less effective, in order to reduce the number of activities incorrectly identified as malicious. Too many errors in the system can exasperate the users (46) to the point that they will circumvent the security features.

Setting up computers and devices to automatically save backups is another strategy frequently employed in hospitals. Creating data backups will not necessarily save a hospital from losing its files, though. Most malwares that delete primary sets of data are set to also delete backups (108).

#### 3.4.2. Financial measures

An estimated 33% of hospitals obtain cybersecurity insurance (6). Insurance will certainly help with the financial effects of an attack after the fact, but it does nothing to prevent, mitigate, or manage an incident.

#### 3.4.3. Device requirement measures

In order to mitigate attacks on or *via* medical devices, most hospitals require at least a few basic specifications to be incorporated. These include information privacy, trustworthiness, validity, and accessibility. The main strategies to attain these standards are encryption, checksum verification, access restrictions, and credential requirements (5). Yet, these do not necessarily mean data is truly secure. For instance, malicious parties may be able to decrypt the files or take the files even while they are still encrypted. Checksum, access restrictions, and credential requirements can similarly be bypassed by skilled attackers with minimal difficulty (104).

#### 3.4.4. Detection and response measures

Per industry professionals, particularly the Society for Imaging Informatics in Medicine, cyber defenders—the ones responsible for mitigating and responding to attacks—must inspect devices, networks, user activities, and security plans; verify and credential the hospital systems' users; ensure systems are functioning properly; and safeguard privacy and soundness of patient records (32).

In healthcare, the cyber defenders are usually the IT department (23), creating two interrelated problems. First, the lack of adequately-sized IT teams means not all of these necessary functions will be achieved. While 75% of hospital cyber incidents were detected by IT departments, 57% of attacks required other employees' involvement in order to be detected, 21% were found by third-party consultants and, troublingly, 5% were found by patients (27). When an attack occurs, only 55% of hospitals have automatic procedures to deploy a response. This is less than the 59% average of all other industries (56). As expressed earlier, hospital IT teams simply do not have the resources to sufficiently protect operations.

The second problem, IT teams sometimes get caught up in preventing cyberattacks. They consequently neglect to address the functional IT issues, like non-security-related software or hardware malfunctions. This can become a dispute between the IT department and healthcare providers, who need computers and equipment to work well (32). At some point, if the balance emphasizes too much the cybersecurity aspects of technology, employees will bypass the protective features (46).

#### 3.4.5. Regulatory measures

Many hospitals rely on government regulations and guidelines to inform their cybersecurity practices. For instance, per HIPAA regulations, each hospital must designate a data security officer, regularly perform risk assessments, and have incident response plans prepared (94). Nevertheless, regulations can be too basic to effectively protect hospitals, like the Centers for Medicare and Medicaid Services mandating only simple antivirus and antimalware tools for hospitals using their services (60, 109).

Alternatively, regulations may be convoluted and possibly contradictory. There is no one agency that oversees healthcare cybersecurity (12). Rather, various divisions of healthcare, each of which contains cyber technologies in some way, are supervised by a variety of agencies, on the state and federal levels. The complex web of divisions makes regulating and synchronizing cybersecurity protocols difficult (92).

In 2018, the National Institute for Standards and Technology created a unified framework that set guidelines for organizations involved in critical infrastructure as to how to detect, evaluate, and handle security protocols and events (60). Despite the strong effort, this framework was taken on by only 58% of hospitals (110). There is still no consensus regarding which framework to use.

There is also no consensus regarding which authority to rely on for notifications about present threats. Of more than a dozen authorities listed, the two highest rated were the Cybersecurity and Infrastructure Security Agency, which was relied on by 60% of hospitals, and the non-profit Healthcare Information and Management Systems Society, which was the choice for 54%. Significantly, 69% reported their primary source of information was colleagues and “word of mouth” (110).

The uncoordinated landscape of cyber-regulating agencies is exaggerated when a hospital is hit by a cyberattack. In addition to HIPAA penalties, the hospital must deal with investigations and penalties from a slew of non-healthcare-specific agencies, such as the Federal Trade Commission (FTC) and the Security and Exchange Commission (SEC) just to name a few. The FTC actively pursues companies that do not protect consumers' cybersecurity, and the SEC mandates transparent reporting of data breaches and cyber threats (28), neither of which has specific jurisdiction over healthcare practices.

Lack of coherence can actually impede adherence to cyber regulations. For instance, smaller hospitals and device manufacturers may not have adequate resources or drive to implement cyber protocols. However, certain legislations limit larger hospitals and companies from assisting them with resource provision (92). The Stark Law, for example, prohibits physicians from referring patients with government health insurance to medical services with which the doctors have financial relationships (111). Hospitals may therefore prefer not to build financial relationships with other facilities (92). They instead may keep resources and data to build their own security programs. Eventually, this can lead hackers to avoid well-protected hospitals and instead target the smaller hospitals.

#### 3.4.6. Gaps highlighted during the pandemic

The COVID-19 pandemic brought to the forefront further gaps in hospitals' cyber preparedness, demonstrating the insufficiency of current protective measures. Cyberattacks rose during 2020, particularly ones involving ransoms (25): hackers targeted a Czech hospital, UK vaccine trial, US health agency, UK emergency COVID-19 hospital construction team, and US, UK, and Canadian vaccine development labs, just to name a few (15). The attacks became so prevalent that governments and the international policing agency INTERPOL (15) released alerts regarding the threats. In the US, cybercriminals compiled a list of more than 400 vulnerable hospitals to target and attacked quite a few (59).

Myriad cybersecurity gaps are presented in the literature to explain this uptick. First, telemedicine platforms used during COVID-19, like Zoom and Skype, do not incorporate end-to-end encryption and are overall usually not as secure as hospitals' networks need them to be (55). Personal devices, which became rampant as employees worked from home or worked from hospitals where not enough telemedicine devices were available, are often unprotected or vulnerable (55). VPNs are generally hospitals' main line of defense for data protection on non-official computers (13), yet VPNs only cover data in-transit.

COVID-19 also exposed further susceptibilities in hospital security associated with internal actors. For individuals, a study found that 22-30% of people had increased “fear,” “hope,” or “relief,” as the health crisis progressed (112). Attackers played on these feelings to more easily deceive patients or providers into fraud (112). For healthcare workers, increased stress plus new technologies and health situations led to cyber mistakes, such as inadvertently clicking on phishing links (13). Moreover, the pandemic increased providers' workloads (55); increased workloads are associated with less attention to cybersecurity protocols (113).

Third, the need for urgent patient health data access increased, which meant hospitals were more willing to pay ransoms to cybercriminals (55). Medical equipment, too, was urgent to obtain as supplies dwindled. Hospitals' frantic searches for personal protective equipment, for example, left them more vulnerable to scams (13).

A last gap that likely contributed to increased cyberattacks was the lack of preparedness on the part of hospital management. Senior management did not fully understand the cyber threat implications, and business continuity plans for events like this were lacking (13). Cyberattacks changed to reflect these new COVID-19-induced vulnerabilities, yet hospitals were not prepared to quickly recognize attacks, proactively mitigate them, or train employees to use new technologies properly or recognize new attack methods. As the hospitals were overwhelmed with critically ill patients, cybersecurity teams received less resources, funding, and attention (13).

## 4. Discussion

### 4.1. Analysis and conclusion

One of the main trends established through comparison of all available, recent, and relevant literature is that serious gaps are present in hospitals' approaches to technical, educational, policy, and resource-allocation elements of cybersecurity. Cybercriminals can access, steal, block, or manipulate screening tools, medication treatments, vital sign alarms, patient records, telecommunication, or clinical supplies, just to name a few. Yet, hospitals lack adequate protections for each of these vulnerabilities. From ineffective measures to advanced innovations that focus on patient care and that neglect the risks such technologies create, to a lack of employee awareness of security protocols, to simply deficient cybersecurity IT teams, hospitals are no match for savvy, and sometimes even amateur, hackers. The research question, whether US hospitals are prepared for cyber risks that accompany clinical medicine, can thus be answered with a succinct “not yet.”

The first step in attack prevention is being aware of the risks (31), as this paper aimed to do. In fact, 69% of hospitals do recognize that healthcare is more at risk of incidents than other industries (6). However, it is apparent that efforts to manage these risks are misdirected. Seventy-nine percent of the most-cited healthcare cybersecurity research topics relate to technology, rather than human-, organizational-, and business-related topics. Only 17% of the most widely-published studies were included in health journals, the other 83% in engineering journals (2). Attention is being pointed toward technical, non-medical-specific vulnerabilities, rather than focusing on the unique root causes and vulnerabilities that hospitals carry.

At the same time, some vulnerabilities simply cannot be prevented. For instance, attackers can gather data from employees to then infiltrate networks or extort the employees. One piece of information that must be clearly distinguished is employees' ID tags, as identifying members of the medical team is key to patient care. However, an experiment showed that gathering employee information simply by seeing their IDs allowed the researchers to search the internet and target them online (9). Such employee information cannot practically be kept confidential. There must be a balance between cybersecurity measures and realistic prevention.

In 2015, the US Congress established a task force to determine root causes for weak cybersecurity among hospitals. They found little collaboration among institutions regarding threats, incongruous cyber protection efforts among healthcare executives, incongruous government regulations, inattention of hospital staff to protocols, inconsistent patient care due to hospitals' operational differences, and inadequate resources allotted to cybersecurity programs (92). Congress again convened a task force in 2017 to design policies based on the findings (114). As shown in this paper, however, many of these findings are still areas of concern in the field.

Perhaps the most crucial takeaway is that when cyberattacks occur in hospitals, patients are the real victims (57). The majority of confidential data that hackers compromise belongs to patients, and it is their health on the line when medical devices are manipulated, hospital computers are rendered inoperable, or treatments are inaccessible. If not for financial, reputational, or functional reasons, then at least for the sake of their patients, hospitals should actively work to prepare themselves for the inevitable cybersecurity risks.

Follow-up studies should discuss lessons that can be taken for healthcare from other industries' cybersecurity methods and, as cybersecurity relies on cooperation of providers and patients, should examine security measures more specifically from provider and patient perspectives.

### 4.2. Policy recommendations

#### 4.2.1. Training

Most of the 35 articles selected mentioned training as a key to better cyber practices (5, 12, 13, 19, 25, 27, 32, 35, 52). Training staff in cybersecurity principles has been shown to reduce the number of attacks (115). Tactics, like social engineering, are constantly changing, so employees need to be regularly updated on best practices. The education should even become part of the hospital culture and strategies (52, 57). Some researchers have been pushing for training of students, wherein future medical providers learn regulations and their roles in cyber protections before they are thrown into the cyber-vulnerable industry (103).

#### 4.2.2. Technical

Updating and patching device and network software can help protect vulnerabilities (116), but care must be taken not to cause harm to patients while their devices undergo updates. Updates can be bundled so multiple updates are installed at once (16). Frequent data backups are also crucial (19), following the “3-2-1 rule” of saving 3 backups of high-priority data, using 2 separate forms of media, and with 1 set saved offline (59). Basic technical measures like antivirus (94), firewalls (32), VPNs (15), encryption (13), multi-factor authentication (35), user activity control including blocking software installations (32), network segmentation (19), digital signatures (19), constant monitoring (32), and physical security of servers (19) are easily procurable commercially (32). However, as mentioned in section “Technical Measures”, they are not sufficiently protective and can make work inefficient, resulting in staff bypassing them (117). Machine learning software, on the other hand, is becoming more popular to detect usually-undetected malwares (46). Also, specialized keys are more effective than basic password requirements (75). Technical measures play a major part in allowing hospitals to avoid paying ransom and should work both defensively and offensively (28).

#### 4.2.3. Risk management

Multiple frameworks for creating risk management plans are proposed in the literature (11, 13, 23, 31, 36, 68, 117, 118), focusing on building resilience, business continuity plans, threat modeling, or various other strategies. The key is to make the rewards of attacking not worth hackers' risk or effort (32).

#### 4.2.4. Group effort

All individuals involved in hospital operations are affected by cyber protocols and events. Thus, representatives from all divisions—executives, floor staff, law enforcement, legal advisors, auditors, other hospitals, etc.,—should inform cybersecurity decisions (28, 33, 65). And all, including contractors and device-makers, should have roles in maintaining cyber safety (59, 92). Strong IT teams, led by dedicated cybersecurity personnel, are a must in the hospital setting (94).

#### 4.2.5. Build into it

Equipment should be acquired only if cybersecurity measures are integrated, not simply added on at the end (65). For example, technologies may have “trust”-based interactions between nodes, wherein a device node learns that information coming from a certain external node is trustworthy (20, 49), or can use blockchains, wherein data is logged in “blocks” to protect data integrity and reliability (17, 69, 119). Similarly, hospital culture should incorporate cybersecurity as an integral element, as if a form of the medical principle “do no harm” (52). Policies for hospitals and manufacturers can be useful in ensuring that technological innovations manage cyber risks (11, 13), thereby reducing the aforementioned “cyberthreat paradox” (100).

### 4.3. Limitations

This study was conducted *via* literature search of academic databases. As such, only cybersecurity issues that have been previously studied or considered in an academic setting were explored in the study, precluding the examination of issues not previously introduced into the academic realm or of less interest to cyber professionals. Further, only *PubMed* and snowballed articles were included, potentially excluding articles solely on other databases.

The articles on which this report is based carried their own limitations, which transferred to this study. Commonly, except for specifically mentioned numbers, such as statistics, most articles were written qualitatively. The results of this study were then considered only qualitatively. Thus, while the study attempted to describe topics according to their weight in the literature (e.g., exploring with greater depth the topics identified by other researchers as higher priorities), some topics were described on more equal prioritization footing or perhaps attributed less significance than deserved.

Lastly, the constantly changing nature of cybersecurity means that although effort was made to include only “recent” articles, some topics may have experienced further innovation since then. Similarly, there may be concerns in the field that are too novel or challenging to be described in literature. Such topics would not have been included in this review.

## 5. Summary

Healthcare is facing a growing threat of cyberattacks. Myriad data sources illustrate the same trends of healthcare being one of the industries with the highest risk of cyber infiltration and seeing the rate of security incidents surge within just a few years. The circumstances thus begged the question: are US hospitals prepared for the risks that accompany clinical medicine in cyberspace?

By studying 35 journal articles, this paper worked to identify the major topics and concerns present in today's hospital cybersecurity field. The basic landscape was depicted by assessing the primary reasons hospitals are frequent targets (financial, political, personal enjoyment, revenge, and white hat purposes), the top ten methods of attack (phishing, man-in-the-middle, malware, drone attacks, etc.), and the consequences hospitals face following attacks (loss of data, money, reputation, patient trust, and safety).

The cyber technologies common in clinical medicine and their risks were then detailed. The major categories highlighted were medical devices, such as MRI machines, insulin pumps, and more; telemedicine software, wherein healthcare providers communicate with other providers or patients *via* often-unsecured portals; and electronic data, which carries risk in its storage and exchange. By infiltrating any of these components of clinical care, cyber-attackers can access a trove of valuable information and manipulate, steal, ransom, or otherwise compromise the records, or can use the access to catapult themselves to access other parts of a hospital's network.

Secondary issues that amplify the cyber risks associated with devices, telemedicine, and electronic data were then explored: interoperability, unprotected operating systems, lack of cyber resources, a focus on medical care over cyber efforts, rapid innovation, and perpetual network availability, to name a few.

Following the identification of the technologies and risks most commonly noted in the journal articles, strategies hospitals tend to employ to combat the risks were identified. This included technical, financial, detection and response, device requirement, and regulatory measures. Such strategies, however, were shown in the literature to be subpar. There exist within the measures serious vulnerabilities and gaps that many of today's hospitals fail to address. To illustrate, gaps still present during the COVID-19 pandemic were discussed.

**Figure 2:**
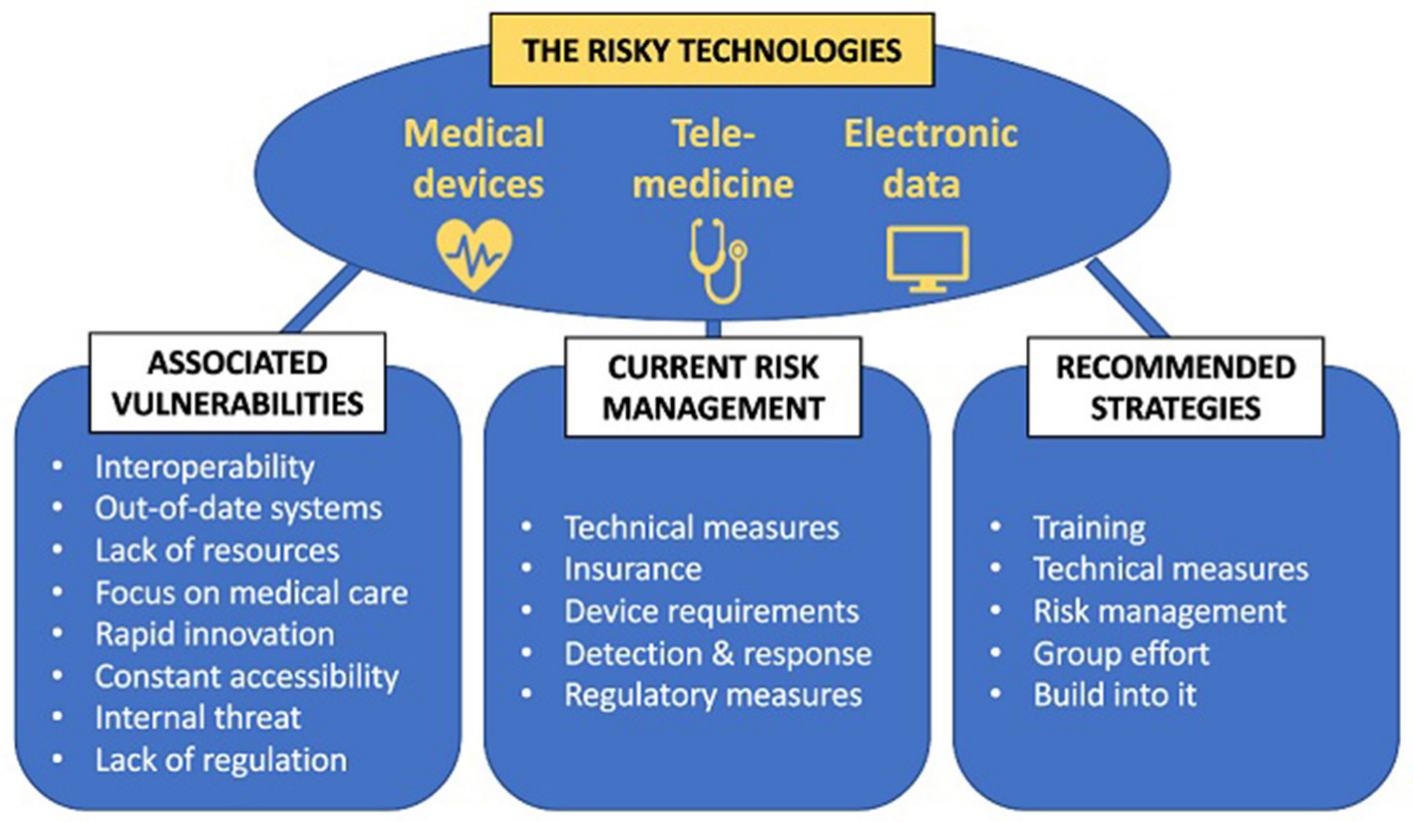
Summary of key findings.

Comparison of the risks, strategies, and gaps revealed that many hospitals in the US are unprepared for cybersecurity risks. The focus of their efforts are misdirected, with external—often governmental—efforts negligible. Several policy recommendations were presented to better combat the gaps, including but not limited to training employees in cyber protocols, adding advanced technical protections, and collaborating with a variety of experts (see Figure 2 for summary diagram).

Overall, hospitals must recognize that, in cyber incidents, the real victims are the patients. They are the ones at risk, physically and in information confidentiality, when medical devices, hospital equipment, or treatments are compromised.

## 6. Key definitions

*Cybersecurity* = the preservation of the integrity and functions of technologies connected to computer servers, safeguarding against unauthorized infiltration or interference, intentional or not, in the technologies' software, hardware, or networks (60).

*Security incident* or *cyber event* = an occurrence in which technology connected to computer servers experiences infiltration or interference by an unauthorized party, successfully or not (120).

*Cybercrime* or *cyberattack* = an unlawful activity carried out *via* computers or technologies connected to computer servers, often by infiltrating or interfering with the systems in an unauthorized manner. In cyber-based crimes, perpetrators can more easily erase evidence that the crimes took place (32).

*Hacker* or *Cyberattacker* = an individual, working independently or with others, who utilizes knowledge of vulnerabilities, skills of network infiltration, and/or an internet connection to infiltrate and/or interfere with computer systems, whether for malicious or benign purposes.

*Vulnerability* = weakness or error in a computer technology's code, usually in its operations or protections, that can be exploited by hackers to infiltrate or interfere with the code (121).

*Data breach* = a cybersecurity incident in which confidential information is exposed, manipulated, stolen, or compromised.

*Darknet* = a “hidden” and deeper realm of the internet which requires certain authorizations or codes in order to access it; the darknet has a reputation, though not always accurate, of hosting unlawful activities (122).

## Author contributions

Both authors listed have made a substantial, direct, and intellectual contribution to the work and approved it for publication.

## Conflict of interest

The authors declare that the research was conducted in the absence of any commercial or financial relationships that could be construed as a potential conflict of interest.

## Publisher's note

All claims expressed in this article are solely those of the authors and do not necessarily represent those of their affiliated organizations, or those of the publisher, the editors and the reviewers. Any product that may be evaluated in this article, or claim that may be made by its manufacturer, is not guaranteed or endorsed by the publisher.
