# Supplementary material for: Hospital cybersecurity risks and gaps: Review (for the non-cyber professional)
Source: Front Digit Health. 2022 Aug 11;4:862221. doi: 10.3389/fdgth.2022.862221 (PMC9403058; doi:10.3389/fdgth.2022.862221)
Supplement: Supplementary file 1 [file Table_1.DOCX]

**Supplemental Materials**

*Table 1.* Data abstraction of the 35 articles selected for this study, with key details listed according to chronological-alphabetical order of the articles’ titles.

| *Table 1: Articles Selected for Development of Main Arguments* | | | |  |
| --- | --- | --- | --- | --- |
| Year | Title | Lead Author | Journal | Ref # |
| 2016 | Cyber threats to health information systems: A systematic review | Luna | *Technology and Health Care* | 24 |
| 2016 | Ransomware in hospitals: What providers will inevitably face when attacked | Kelpsas | *The Journal Of Medical Practice Management: MPM* | 28 |
| 2017 | Cyberattack on Britain's National Health Service - A wake-up call for modern medicine | Clarke | *The New England Journal of Medicine* | 14 |
| 2017 | Cybersecurity and healthcare: How safe are we? | Martin | *BMJ* | 11 |
| 2017 | Cybersecurity in healthcare: A systematic review of modern threats and trends | Kruse | *Technology and Health Care* | 1 |
| 2017 | Cyber-security issues in healthcare information technology | Langer | *Journal of Digital Imaging* | 32 |
| 2017 | Holding the line: Events that shaped healthcare cybersecurity | Grimes | *Biomedical Instrumentation & Technology* | 3 |
| 2017 | The role of healthcare technology management in facilitating medical device cybersecurity | Busdicker | *Biomedical Instrumentation & Technology* | 58 |
| 2018 | Cybersecurity in healthcare: A narrative review of trends, threats and ways forward | Coventry | *Maturitas* | 12 |
| 2018 | Healthcare blockchain system using smart contracts for secure automated remote patient monitoring | Griggs | *Journal of Medical Systems* | 69 |
| 2019 | Are implanted electronic devices hackable? | Alexander | *Trends In Cardiovascular Medicine* | 27 |
| 2019 | Assessment of employee susceptibility to phishing attacks at US health care institutions | Gordon | *JAMA Network Open* | 35 |
| 2019 | Cryptopharmaceuticals: Increasing the safety of medication by a blockchain of pharmaceutical products | Nørfeldt | *Journal of Pharmaceutical Sciences* | 17 |
| 2019 | Cybersecurity and privacy issues for socially integrated mobile healthcare applications operating in a multi-cloud environment | Al-Muhtadi | *Health Informatics Journal* | 20 |
| 2019 | Cybersecurity features of digital medical devices: An analysis of FDA product summaries | Stern | *BMJ Open* | 76 |
| 2019 | Cybersecurity in cardiac implantable electronic devices | Martignani | *Expert Review of Medical Devices* | 5 |
| 2019 | Health care and cybersecurity: Bibliometric analysis of the literature | Jalali | *Journal of Medical Internet Research* | 2 |
| 2019 | Intelligent and dynamic ransomware spread detection and mitigation in integrated clinical environments | Fernández Maimó | *Sensors (Basel)* | 46 |
| 2019 | Phishing in healthcare organisations: Threats, mitigation and approaches | Priestman | *BMJ Health & Care Informatics* | 9 |
| 2019 | Protecting patient data is the new scope of practice: A recommended cybersecurity curricula for healthcare students to prepare for this challenge | Swede | *Journal Of Allied Health* | 103 |
| 2020 | Cyber attacks on healthcare devices using unmanned aerial vehicles | Sethuraman | *Journal of Medical Systems* | 49 |
| 2020 | Cybersecurity in PACS and medical imaging: An overview | Eichelberg | *Journal of Digital Imaging* | 19 |
| 2020 | Cybersecurity risks in a pandemic | Williams | *Journal of Medical Internet Research* | 55 |
| 2020 | Cybersecurity update: Recent ransomware attacks against healthcare providers | Dullea | *Missouri Medicine* | 59 |
| 2020 | Healthcare challenges in the era of cybersecurity | Tully | *Health Security* | 60 |
| 2020 | Healthcare cyber-attacks and the COVID-19 pandemic: An urgent threat to global health | Muthuppal- aniappan | *International Journal for Quality in Health Care* | 15 |
| 2020 | Information security climate and the assessment of information security risk among healthcare employees | Kessler | *Health Informatics Journal* | 8 |
| 2020 | “It's like a cyber-security blanket": The utility of remote activity monitoring in family dementia care | Mitchell | *Journal of Applied Gerontology* | 67 |
| 2020 | Risk management-based security evaluation model for telemedicine systems | Kim | *BMC Medical Informatics and Decision Making* | 21 |
| 2020 | Transforming healthcare cybersecurity from reactive to proactive: Current status and future recommendations | Bhuyan | *Journal of Medical Systems* | 23 |
| 2020 | Why employees (still) click on phishing links: Investigation in hospitals | Jalali | *Journal of Medical Internet Research* | 113 |
| 2021 | Cybersecurity threats to cardiac implantable devices: Room for improvement | Tomaiko | *Current Opinion in Cardiology* | 75 |
| 2021 | Health care cybersecurity challenges and solutions under the climate of COVID-19: Scoping review | He | *Journal of Medical Internet Research* | 13 |
| 2021 | Maybe if we turn it off and then turn it back on again? Exploring health care reform as a means to curb cyber attacks | Farringer | *Journal of Law, Medicine & Ethics* | 92 |
| 2021 | Medical apps and the gray zone in the COVID-19 era: Between evidence and new needs for cybersecurity expansion | Maccioni | *Healthcare (Basel)* | 18 |
